# From the literature

**Published:** 2008

**Authors:** Nandini AS

**Affiliations:** *Dermatologist, Venkat Charmalaya Centre for Advanced Dermatology, Bangalore, India*

## Pigmentation Due to Stasis Dermatitis Treated Successfully with a Non-coherent Intense-Pulsed Light Source

**Pimentel CL, Rodriguez-Salido MJ.**

Dermatol Surg. 2008 Jul;34(7):950-1

Ochre dermatitis is a secondary pigmentary disorder of venous stasis in chronic venous insufficiency where the increase in intravascular pressure and endothelial alterations causes extravasation of erythrocytes, haemosiderine-laden macrophages and melanin deposits. A 69-year-old Caucasian female with pigmentary ochre dermatitis presented with purpuric macules and patches, ochre-brownish in colour, on the lower third of both legs with no discomfort expect her aesthetic problem. Three sessions of non-coherent intense-pulsed light (IPL) were performed with the following parameters: 570 nm, 15 ms, 10–12 J/cm, after which lesions initially darkened and then lightened, becoming skin coloured completely, after a few weeks. No repigmentation was observed after a 6-month follow-up. No side effects occurred. The study demonstrates the possible efficacy of IPL in stasis dermatitis, which is otherwise difficult to treat. However, the finding needs to be confirmed in larger studies.

## Fordyce Spots of the Lip Responding to Electrodesiccation and Curettage

**Chern PL, Arpey CJ.**

Dermatol Surg. 2008 Jul;34(7):960-2

Fordyce spots are heterotopic sebaceous glands typically located on the vermilion lip or the oral mucosa. A 46-year-old male presented for enlarging whitish-yellowish 1–2 mm papules on the upper vermilion lip. These were of significant cosmetic concern to the patient. The papules were most prominent with smiling, causing him distress at work and in his social interactions. Electrodesiccation and curettage was performed as it was the simplest, most affordable and lowest risk approach. A total of four treatments spaced 4–6 weeks apart, with elctrodesiccation with a hyfrecator set within the high energy range but a numerical value of 4, a relatively low setting, for approximately 1 s or less until a light char was seen, followed by one pass with a damp 4×4 gauze and gentle curette to express exposed gland lobule material. Mild scale and minimal crusting was seen for 1 week. No complications or scarring occurred. Complete resolution of treated Fordyce spots was seen after four treatments without any recurrence for 1 year after the initial treatment.

## Subungual Pyogenic Granuloma Treated by Sodium Tetradecyl Sulphate Sclerotherapy

**Moon SE.**

Dermatol Surg. 2008 Jun;34(6):846-7

Pyogenic granuloma is a relatively common, acquired haemangioma composed of well-differentiated vessels. Sometimes it is unclear which treatment approach to use. This is especially the case for lesions in the subungual location. A 33-year-old woman presented with a well-circumscribed 4 mm, pedunculted, dark red-coloured subungual papule of 1 month duration on her right thumb. Sodium tetradecyl sulphate 0.5% solution was injected slowly into the mass until blanching was observed. After 1 week, the tumour shrunk considerably, leaving a small residual lesion and the second injection was performed. At 1 week after the second treatment, the papule disappeared completely. The total volume of solution injected was 0.1 ml. The patient did not complain of any pain and there was no adverse reaction. No recurrence was noted 1 year after treatment. Although sclerotherapy requires a couple of treatment sessions, it is a simple, convenient and economic method. It does not require anaesthesia or expensive equipment such as laser.

## Dermabrasion for Actinic Cheilitis

**Dufresne RG Jr, Cruz AP, Zeikus P, Perlis C, Jellinek NJ.**

Dermatol Surg. 2008 Jun;34(6):848-50

Actinic Cheilitis (AC) is a common pre-malignant condition of the lips resulting from chronic or excessive ultraviolet exposure. Current treatment methods include topical 5-fluorouracil, imiquimod, photodynamic therapy, chemical peels, cryosurgery, electrodesiccation and curettage, carbon dioxide laser and vermilionectomy with or without mucosal advancement. Four patients with SCC of the lower lip were initially treated with Mohs surgery. After clearing the tumour, the vermilion was subsequently dermabraded and feathered onto the cutaneous surface. One or two passes were used with a fine pear-shaped diamond fraise until a dull appearance was noted on the vermilion. The technique was quick and easy. Reepithelialization occurred by 2 weeks. No clinical recurrence of AC or SCC was seen at the 6-month follow-up visit. Dermabrasion is readily available and is quick and easy to perform, with limited morbidity.

## Calcinosis Cutis of Juvenile Dermatomyositis Treated with Incision and Drainage

**Wu JJ, Metz BJ.**

Dermatol Surg. 2008 Apr;34(4):575-7

A 17-year-old Caucasion male with a long-standing history of juvenile dermatomyositis presented with calcinosis cutis of the fingers presenting as multiple white and erythematous subcutaneous nodules over the distal and proximal interphalangeal joints. No slowing of progression of calcinosis was seen despite systemic steroids. A no. 11 blade was used to incise the lesion at the tip of the finger, which was soft and fluctuant. Firm pressure was placed distal and proximal to the incision and a thick, white, chalky material was extruded. Patient reported marked improvement in pain and movement of that digit immediately after the procedure.

## The Use of Sterile Gauze as a Retractor When Performing Surgery in the Web Spaces

**Harting M, Orengo I.**

Dermatol Surg. 2008 Apr;34(4):583

Biopsies or excisions in the web spaces of the hands or feet are generally thought be cumbersome due to difficulty in visualization. The assistant’s hands present as an obstruction between the surgeon and the surgical site, given the small size of the lesions in consideration. A 4×4 cm sterile gauze pad is unfolded, laid out flat and is then twisted to form a rope-like structure, which is then placed within the web space, and gentle retraction is applied medially and similarly laterally. In addition to providing visualization, this technique also allows the assistant to maintain a safe distance between his or her hands and any sharp instrument. If sterile gauze pads are not available, this technique can be performed using rolled sterile gauze, sterile self-adhering wrap dressing or an extra pair of surgical gloves.

